# Factors associated with quality of care in inflammatory bowel diseases: a view from patient’s side using the IQCARO quality of care decalogue

**DOI:** 10.1186/s12876-021-01968-2

**Published:** 2021-10-29

**Authors:** F. Casellas, Xavier Calvet, D. Carpio, I. Vera, R. Saldaña, M. Mínguez, L. Marín, B. Juliá, GETECCU, GETEII and ACCU

**Affiliations:** 1 Digestive Diseases Unit, Corporació Sanitaria Universitària Parc Taulí, Parc del Taulí, 1. 08208 Sabadell, Barcelona, Spain; 2 Digestive Diseases Unit, Corporació Sanitaria Universitària Parc Taulí, Sabadell, Barcelona Spain; 3 Departament de Medicina, Universitat Autònoma de Barcelona, Bellaterra, Spain; 4 CIBEREHD, Instituto de Salud Carlos III, Madrid, Spain; 5 Complexo Hospitalario Universitario de Pontevedra, Instituto de Investigación Biomédica Galicia Sur (IBI), Pontevedra, Spain; 6 Servicio de Aparato Digestivo, Hospital Universitario de Puerta de Hierro, Majadahonda, Madrid, Spain; 7 ACCU, Madrid, Spain; 8 Hospital Clínico Universitario, University of Valencia, Valencia, Spain; 9 Servei de Malalties Digestives, Hospital Germans Trias I Pujol, Barcelona, Spain; 10 Medical Department MSD, Madrid, Spain

**Keywords:** Quality of health care, Inflammatory bowel diseases, Surveys and questionnaires, Ulcerative colitis, Crohn’s disease, Patients

## Abstract

**Background:**

Quality of care (QoC) is a highly important topic in inflammatory bowel disease (IBD). We recently elaborated a decalogue of QoC indicators (IQCARO-QoC) developed by IBD patients. The aim of the present study was to assess the factors associated with patients’ evaluation of QoC in Spain using the IQCARO-QoC Decalogue recently developed by IBD patients.

**Methods:**

A survey including patients’ socio-demographic and clinical characteristics, and the IQCARO-QoC Decalogue, was completed by IBD patients. We described patients’ assessment of QoC across Spanish patients. A univariable and multivariable analysis was performed to explore the associations between patients’ characteristics and QoC.

**Results:**

Questionnaires from 788 participant patients were analysed. Participants’ mean age was 43.4 years, 63% were females and 58% had Crohn’s disease. The mean QoC score was 8.1 (± 2.4 SD) points out of a maximum of 10. Items with the lowest score were related to the provision of information and the implication of the medical team throughout the entire patient care. Factors associated with better QoC scores included: being employed better disease control, fewer numbers of unscheduled visits, and being followed by a gastroenterologist specialized in IBD.

**Conclusions:**

Spanish patients’ reported QoC seems to be globally good although there is room for improvement, especially in providing adequate information to patients. Care provided by specialized IBD gastroenterologists seems to be related with higher QoC scores.

## Background

Inflammatory bowel diseases (IBD), such as Crohn’s disease (CD) and ulcerative colitis (UC), are chronic and relapsing inflammatory disorders that are frequently diagnosed early in life and require long-term healthcare [1]. The global management of IBD is complex. Patients need clinical and laboratory follow-up regularly, multiple endoscopic and radiological evaluations and, often aggressive treatment, even including surgery. Multidisciplinary teams involving gastroenterologists, surgeons, pathologists, radiologists and nurses are often necessary to provide adequate care [2].

Health-related quality of care (QoC) has recently drawn increased attention in IBD, with various international guidelines and quality indicators being developed to assess every aspect of health care delivery [2, 3]. The World Health Organization (WHO) definition of QoC is “the extent to which health care services provided to individuals and patient populations improve desired health outcomes. In order to achieve this, health care must be safe, effective, timely, efficient, equitable and patient-centered” [4, 5]. In this context, patients’ perspective is increasingly being recognized as a necessary element for health care evaluation [3]. Recently, the WHO suggested that both clinical and patients’ perception of QoC need to be collected and compared for a comprehensive and detailed assessment of the quality of health services [6]. However, at present, tools to measure QoC in IBD from the patients’ perspective are complex [7] and are barely applied to the clinical practice [8–10].

Our group has recently developed and published the IQCARO Decalogue of QoC indicators (IQCARO-QoC Decalogue) [11], a simple questionnaire to evaluate the QoC from patients’ perspective. This questionnaire consists of 10 critical items related to the management of IBD that have been selected by the patients themselves. The Decalogue, whose completion is facilitated by the use of a dichotomous (yes/no) formulation, was developed in order to be easily understood and completed by any patient, independently of their educational background [10]. In the IQCARO Phase II, we have measured the QoC reported by patients with IBD across Spain using the abovementioned decalogue and have found that higher QoC measured with this tool has been shown to be related to better IDB outcomes [12].

The aim of this study was to assess the QoC reported by IBD patients across Spain using the IQCARO-QoC Decalogue and to describe the factors associated with patients’reported QoC.

## Methods

The present project follows the STROBE 2007 (v4) Statement for reporting cross-sectional studies [13].

### Study design

The IQCARO phase II project consisted of an observational study based on a cross-sectional survey.

### Settings

The study had four sources of participant patients with the aim of incluiding the widest spectrum of IBD patients independently of the type of disease (Crohn´s disease or Ulcerative Colitis) severity of the disease, treatment received or health care setting. On one side, the survey was distributed on paper to sixty IBD Units from secondary and tertiary hospitals across Spain selected by the Spanish Working Group on Crohn’s Disease and Ulcerative Colitis (GETECCU) with the aim of being representative of all autonomous regions in Spain. The IBD specialists who participated in this study were instructed to distribute the survey to 9 consecutive patients, who attended the clinic routinely, irrespective of their disease severity or any other criterion, in order to minimise the selection bias. The patients completed the survey in their homes and returned it by prepaid postal mail. Secondly, posters were placed in the waiting rooms across all the included IBD Units inviting aditional patients to participate. These posters included a QR code redirecting to the online survey. In addition, a link to the survey was posted in the website of the Confederation of Spanish Associations of Patients with Crohn’s Disease and Ulcerative Colitis (ACCU), and finally, ACCU sent e-mails to all their members containing a link redirecting to the online survey. All surveys were completed anonymously and voluntarily by patients.

### Participants

The sample consisted of patients with IBD distributed throughout Spain. Participants voluntarily completed the survey that they received from their treating physicians, on paper, or online after scanning the QR code on the waiting room or through the ACCU web page. From October to December 2017, patients diagnosed with CD or UC, regardless of the severity of the disease or the type of treatment, were recruited. Patients under 18 years of age or those unable to understand or complete the survey were excluded. All patients provided informed consent to participate in the study.

### The survey instrument

The IQCARO II project survey comprised of two questionnaires with a total of 27 questions, which were self-administered by the patients. The first questionnaire included 17 questions about demographic and clinical characteristics as well as major IBD outcomes (surgical procedures, ostomies, unscheduled visits, hospitalisations, disease activity in the preceding year, disease control in the preceding two weeks, number of flares in the last year, among others). Full details of the survey can be found elsewhere [14]. The second questionnaire included the 10 indicators from the IQCARO-QoC Decalogue [11] and was provided in the form of a dichotomous (yes/no) formulation (Table 1). Based on the answers to the decalogue we constructed a simple QoC index (QoCI). The QoCI ranged from 0 to 10 points (0 = worst, 10 = best), resulting from the sum of the “yes” answers to each item of the decalogue.

**Table 1:** IQCARO QoC decalogue

	QoC indicator definition
1	My IBD care team has provided me with enough information about my illness
2	The medical team that manages my illness participates in all phases of care (emergencies, outpatient consultation, hospitalization, endoscopy, etc.)
3	My doctor pays me proper attention during my medical appointment
4	In case of an emergency, I can reach urgently my IBD care team when I have symptoms of an outbreak or complication
5	I am convinced that my IBD care team is capable to handle my illness correctly
6	My opinion, my personal and work situation have been taken into account when making decisions about the management of my illness
7	When I go to the outpatient clinic or hospital I have toilets nearby
8	Within my IBD care, I know who the physician in charge of my case is
9	I have been offered recommendations to help me manage my illness in my daily life
10	I have received information about the benefits and risks before starting any treatment for my illness

### Statistical methods

We performed a descriptive analysis of the variables collected. The qualitative variables were summarized by absolute frequencies and percentages, while quantitative variables were described through the mean, standard deviation, median, minimum, maximum and interquartile range.

We carried out an analysis to explore associations between socio-demographic and clinical characteristics of IBD patients and the QoCI obtained after the completion of IQCARO-QoC Decalogue using appropriate statistical tests, according to the nature of the variables analyzed (Chi squared, U Mann-Whitney, Kruskal-Wallis or the Spearman correlation coefficient). Taking into account responses from online questionnaires, we performed a multivariable analysis where the QoCI was dichotomized to be used as a dependent variable in a binary stepwise manual logistic regression model in order to determine the factors that may influence the assessment of a higher QoC (high QoC was defined as 10 points). Significance was set as a two-tailed *p* value of 0.05. The SPSS 19.0 software was used for the statistical analyses.

## Results

### Patients’ demographic and clinical characteristics

**Fig. 1:**
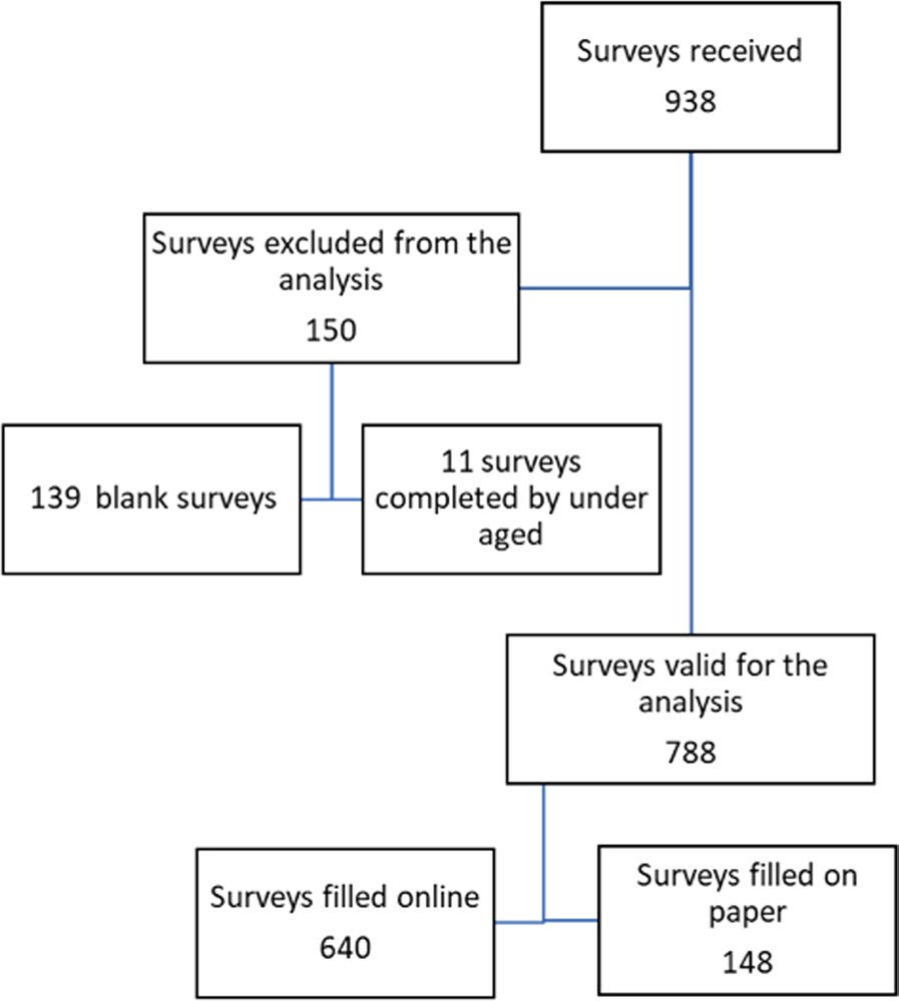
Study flow chart

Nine hundred and thirty-eight surveys were received, 790 of them were answered online and 148 on paper. One hundred and fifty surveys (16%) were excluded from the analysis because they were not filled in correctly (139, 14.8%) or were completed by patients under 18 years of age (11, 1.2%). Therefore, a total of 788 questionnaires were valid for subsequent analysis: 640 online and 148 on paper (Fig. 1).

The mean age of participants was 43.4 years (range: 18–84 years), 63% were females and 58.1% were diagnosed with CD. The mean time since diagnosis was 13 years (range: 1–44 years). The population included patients treated in 183 different medical centers from the 17 Spanish autonomous communities. Demographic and clinical characteristics of the participants are shown in Table 2.

**Table 2:** Demographic characteristics and medical history of the participants

n	788
Mean age ± SD (years)	43.4 ± 12.2
Female/male ratio, n	494/289
Educational background, n (%)	
Primary education	122 (15.6)
Secondary education	53 (6.8)
High school or vocational training	271 (34.6)
Bachelor’s or equivalent level	338 (43.1)
Current employment status, n (%)	
Employed	433 (55.3)
Work disability	106 (13.5)
Unemployed	88 (11.2)
Retired	65 (8.3)
Student	59 (7.5)
Housekeeper	29 (3.7)
Other	3 (0.4)
Type of Inflammatory bowel disease, n (%)	
Crohn’s disease	456 (57.8)
Ulcerative colitis	321 (40.7)
Other (undetermined colitis or unknown)	11 (1.3)
Number of years since diagnosis	
Mean (SD)	13 (9.8)
Median (IQR)	11 (4-20)
Specialist that routinely performed follow-up, n (%)	
Gastroenterologist specialized in IBD	577 (76.6)
General gastroenterologist	160 (21.1)
Surgeon	4 (0.5)
General practitioner	4 (0.5)
Internist	4 (0.5)
Other	5 (0.7)

Percentages do not always add up to 100% because some patient data may be missing

*IBD* inflammatory bowel disease, *IQR* interquartile range, *SD* standard deviation

### Measurement of quality of care index

The mean QoCI score, as measured by the IQCARO QoC Decalogue, was 8.1 (± 2.4) points out of a maximum of 10. The item that scored the highest (9.2 out of 10) was the one questioning whether the patients knew who was the physician in charge of their case. The item that scored the lowest (6.4 out of 10) was the one that enquired whether patients had been offered recommendations to help them manage their illness in their daily life. Specific assessment of each item is shown in Table 3.

**Table 3:** Responses to the IQCARO QoC decalogue

	Definition of the QoC indicators	Number of valid responses	Number and percentage of ‘yes’ answers
1	My IBD care team has provided me with enough information about my illness	744	613 (82.4%)
2	The medical team that manage my illness participate in all stages of care (emergencies, outpatient consultation, hospitalization, endoscopy, etc.)	736	546 (74.2%)
3	My doctor pays me proper attention during my medical appointment	743	660 (88.8%)
4	In case of an emergency, I can reach urgently my IBD care team when I have symptoms of an outbreak or complications	740	593 (80.1%)
5	I am convinced that my IBD care team is capable of handling my illness correctly	737	662 (89.8%)
6	My opinion, my personal and work situation have been taken into account when making decisions about the management of my illness	729	584 (80.1%)
7	When I go to the outpatient clinic or hospital I have toilet facilities nearby	736	661 (89.8%)
8	Within my IBD care, I know who the physician in charge of my case is	744	671 (90.2%)
9	I have been offered recommendations to help me manage my illness in my daily life	739	475 (64.3%)
10	I have received information about the benefits and risks before starting any treatment for my illness	738	552 (74.8%)

Percentages do not always add up to 100% because some patient responses may be missing.* QoC* Quality of care

### Factors associated with quality of care index score

In a univariable analysis, the QoCI was statistically higher in male patients, older participants, patients currently employed, those with longer disease duration, those who were followed by a gastroenterologist specialised in IBD. Similarly, those patients that perceived their disease as inactive in the last year and those that perceived their disease well controlled in the last 2 weeks scored higher in the QoCI (Table 4).

**Table 4:** Relation between demographic and clinical characteristics and the quality index obtained from the IQCARO QoC Decalogue

	n	Quality index – mean (SD)	Quality index – Spearman’s correlation	*p* value
Sex*				0.003
Male	276	8.4 (2.2)		
Female	463	7.9 (2.6)		
Age (years)^#^			0.166	<0.001
Educational background*				0.053
High	312	7.9 (2.6)		
Medium/low	429	8.2 (2.4)		
Current employment status^&^				0.004
Employed	409	8.2 (2.3)		
Work disabled	97	7.9 (2.8)		
Unemployed	87	7.3 (2.7)		
Type of IBD*				0.523
Crohn’s disease	428	8.1 (2.4)		
Ulcerative Colitis	308	8.1 (2.4)		
Number of years since diagnosis^#^			0.116	0.002
Follow-up performed by a digestive specialist*				<0.001
Yes	713	8.2 (2.3)		
No	31	4.5 (3.2)		
Type of doctor that routinely monitors the patient*				
Specialized gastroenterologist	569	8.5 (2.2)		<0.001
General gastroenterologist	153	7.1 (2.7)		
Number of scheduled visits in the last year^#^			0.086	0.024
History of surgery*				0.314
Yes	255	8.2 (2.3)		
No	488	8.0 (2.5)		
Number of surgical interventions^#^			-0.027	0.675
Ostomy *				0.272
Yes	255	8.2 (2.3)		
No	488	8.0 (2.5)		
Self-reported disease activity in the preceding year*				<0.001
Inactive/mild	434	8.5 (2.1)		
Moderate/severe	307	7.5 (7.5)		
Self-reported disease control in the last two weeks^&^				0.01
Well controlled	513	8.8 (1.9)		
Partially controlled	168	6.8 (2.8)		
Poorly controlled	58	5.9 (2.8)		
Self-reported number of flares in the last year^#^			-0.188	<0.001
Number of admissions due to IBD in the last year^#^			-0.082	0.027
Number of emergency/unscheduled visits due to IBD in the preceding year^#^			-0.244	<0.001
Centre size (number of beds)				0.236
≤200	50	7.7 (2.6)		
201–500	170	8.0 (2.5)		
501–1000	321	8.3 (2.3)		
>1000	178	8.0 (2.4)		

Percentages do not always add up to 100% because some patient responses may be missing.

*IBD* inflammatory bowel disease, *QoC* quality of care, *SD* standard deviation

Statistical test used: *Mann-Whitney test; ^&^Kruskal-Wallis test ; ^#^ Spearman’s correlation

We performed a multivariable analysis of the online completed surveys and found that the factors associated to the highest QoCI included the following variables: being employed (OR = 2.974), being treated by a gastroenterologist specialised in IBD (OR = 3.051), have a controlled disease (OR= 2.969 ) and lower number of unscheduled visits (OR = 0.818) (Table 5).

**Table 5:** Participants characteristics related to the highest score in the IQCARO QoC decalogue

	Coefficient	Standard error	OR	95% CI	*p* value
Employment status: unemployed (no)	1.090	0.345	2.974	1.51–5.85	0.002
Treated by specialized gastroenterologist (yes)	1.115	0.247	3.051	1.88–4.95	0.000
Controlled disease	1.088	0.227	2.969	(1.90; 4.63)	0.000
Unscheduled visits	0.201	0.067	0.818	(0.72; 0.93)	0.003
Constant	−2.896				0.000

Binary logistic regression model

*OR* odds ratio, *CI* confidence interval

## Discussion

In this cross-sectional study, patients rated the QoC they received using a simple, self-administered and easy to perform decalogue of critical indicators directly developed by patients. The results of this survey, completed by a significant number of IBD patients (n = 788), suggested that the QoC received in different Spanish centers is good, although there is room for improvement.

A remarkable finding is that there was a strong positive correlation between QoC scores and being treated by a gastroenterologist specialized in IBD. As better IQCARO QoC Decalogue scores were related to improved reported outcomes [12], this finding strongly suggests that IBD patients benefit from being managed by IBD-specialized gastroenterologists.

In addition, there is a positive correlation between higher scores and self-perceived better disease control and, accordingly, negative correlation between higher scores and clinical characteristics that suggest poor control as the number of unscheduled visits.

Despite the unquestionable interest in evaluating QoC from patients’ point of view, until recently, the only objective available tool to assess QoC received by patients with IBD was the QUOTE-IBD questionnaire [7]. This questionnaire consists of 10 generic and 13 IBD specific items and combines importance and performance evaluations. This questionnaire has an indisputable value, but it has barely been used in clinical practice or research probably because it is not easy to complete by the patients in clinical practice. [8–10, 15]. In addition, numerous patient-reported outcome measures (PROMS) have emerged recently, both generic and disease-specific, that underline patients’ experience with the disease and its treatment, including thoughts, impressions, perceptions and attitudes, and are regularly used in IBD clinical trials [3, 16]. However, there is no gold standard to evaluate QoC from patients’ perspective and currently there are no validated assessment tools to measure quality of care in IBD [3].

In line with these efforts to find valued based- and easy to use-tools to evaluate QoC from patients perspective, our group recently developed the IQCARO QoC Decalogue, where IBD patients selected the most important indicators, which, from their perspective, should be taken into account when assessing the quality of IBD healthcare [11]. By using this questionnaire, we detected some areas of improvement when delivering care to IBD patients, including the provision of recommendations to help them manage their illness in their daily life, or information about benefits and risks before starting any treatment. In addition, our results suggest that more implication of the medical team in all stages of the healthcare (emergencies, outpatient consultation, hospitalization, endoscopy, etc.) is necessary. On the other hand, the results suggest that patients know who the physician in charge of their case is, think that their doctors pay them proper attention during their medical appointment, and believe that the IBD care teams are well qualified to manage their illness. These findings are somewhat in line with those from Bortoli et al. who addressed QoC using the QUOTE-IBD questionnaire, in Italy. Responses from 992 patients showed that QoC was rated as satisfactory overall but participants were more critical on aspects related to continuity of care and ﻿﻿﻿information [8]. Similarly, Jelsness-Jørgensen et al. recruited 411 IBD patients from nine hospitals in Norway and, using a purposely-designed 26-item questionnaire, found that patients were satisfied with the QoC received. However, communication seemed to be an important area for improvement, not only between patient and physician, but also among the various healthcare levels [17].

In addition, in our study we wanted to investigate if there is an association between patient characteristics and reported QoC, as measured by the IQCARO QoC Decalogue. In the univariable analysis male sex, older age, longer disease duration, or treated by a gastroenterologist specialized in IBD and in general those with better controlled disease were significantly related with higher IQCARO QoC Decalogue scores. Interestingly, some of these patterns of responses are consistent with previous data obtained in other countries and using different tools. Gonczi et al. evaluated the perceived QoC in 525 IBD patients in Canada and found that female sex, current disease activity, poor health-related-QoC, and poor disease control were all associated with lower quality scores obtained from the QUOTE-IBD questionnaire [9]. Vasudevan et al. [10] analysed the satisfaction with the QoC received by 187 IBD patients in a single centre in Australia, by using also the QUOTE-IBD survey. In the bivariable analyses, they showed that patients exhibiting lower satisfaction were characterized by having worse disease related outcomes such as elevated C-reactive protein, previous bowel resection(s) and by being unemployed. They suggested that these parameters are a surrogate measure of disease-related disability. In this study, longer IBD duration was also associated with lower satisfaction. By contrast, in our study we found a weak correlation between disease duration and higher satisfaction although this variable did not remain significant in the multivariable analysis. As in our study, Vasudevan et al. reported that female sex and younger age were significantly associated with lower QoC scoring. The lower satisfaction of females compared with males was also reported in the original QUOTE-IBD study [7], and is a consistent result of many patients´ satisfaction studies [18–20].

However, to further evaluate the relationship between QoC as measured by the IQCARO-QoC Decalogue and disease outcomes, in the phase II of the IQCARO project we performed a sub-analysis comparing the disease outcomes in patients who reported high QoC (defined as those patients being in the first QoCI quartile) versus patients with poor QoC (those in the 4th QoCI quartile). We found that patients scoring in the first QoCI quartile reported a decreased rate of moderate/severe disease (34.8% vs. 55.3%, *p* < 0.001), fewer number of flares (*p* < 0.001), and less emergency/unscheduled visits (*p* < 0.001) compared with those in the lower QoCI quartile. High QoC group also reported a better disease control [12].

Among the strengths of our study we would like to highlight that it includes a large sample of IBD patients and may offer a credible picture of the QoC delivered to IBD patients from different healthcare settings in Spain. In addition, the fact that the decalogue has been developed by the patients themselves, and that it is easy to use, together with the consistency among our results with those obtained by other studies carried out in different countries and using different tools, also indirectly supports the validity of the IQCARO QoC Decalogue as an instrument for evaluating QoC in IBD patients. However, this study also has some limitations. The inherent limitations of the cross-sectional design of the study preclude any cause-effect interpretation of the associations described. Additionally, the IQCARO-QoC Decalogue is a newly developed tool, which requires further validation with similar instruments or objective measures of QoC, and must be tested in different clinical settings, countries, or regions within the same country. An additional limitation is that sampling was opportunistic, and young and technology skilled patients may be overrepresented, together with the impossibility to determine a response rate, due to the multiple dissemination strategies of the survey.

## Conclusions

In conclusion, by using the IQCARO-QoC Decalogue we observed that the IBD patients reported good QoC across Spain. Areas of improvement were the provision of information and the implications of the medical team in all stages of care. Remarkably, there was a positive correlation between higher QoC scores and receiving care from an IBD specialist.

## Data Availability

The datasets during and/or analysed during the current study available from the corresponding author on reasonable request.

## Abbreviations

ACCU: Confederation of Spanish Associations of Patients with Crohn’s Disease and Ulcerative Colitis
CD: Crohn’s disease
CI: confidence interval
GETECCU: Spanish Working Group on Crohn’s Disease and Ulcerative Colitis
IBD: inflammatory bowel disease
IQR: interquartile range
OR: odds ratio
PROMS: patient-reported outcome measures
QoC: quality of care
QoCI: quality of care index
QR code: quick response code
SD: standard deviation
UC: ulcerative colitis
WHO: World Health Organization
